# Effect of Boric Acid Content in Aluminosilicate Matrix on Mechanical Properties of Carbon Prepreg Composites

**DOI:** 10.3390/ma13235409

**Published:** 2020-11-27

**Authors:** Eliška Haincová, Pavlína Hájková

**Affiliations:** Unipetrol Centre for Research and Education, Revoluční 84, 40001 Ústí nad Labem, Czech Republic; pavlina.hajkova@unicre.cz

**Keywords:** composite, carbon fiber, prepreg, temperature resistant, tensile strength, short-beam

## Abstract

This work presents carbon fabric reinforced aluminosilicate matrix composites with content of boric acid, where boron replaces aluminum ions in the matrix and can increase the mechanical properties of composites. Five different amounts of boric acid were added to the alkaline activator for preparing six types (including alkaline activator without boric acid) of composites by the prepreg method. The influence of boric acid content in the matrix on the tensile strength, Young’s modulus and interlaminar strength of composites was studied. Attention was also paid to the influence of boron content on the behavior of the matrix and on the internal structure of composites, which was monitored using a scanning electron microscope. The advantage of the aluminosilicate matrix is its resistance to high temperatures; therefore, tests were also performed on samples affected by temperatures of 400–800 °C. The interlaminar strength obtained by short-beam test were measured on samples exposed to 500 °C either hot (i.e. measured at 500 °C) or cooled down to room temperature. The results showed that the addition of boron to the aluminosilicate matrix of the prepared composites did not have any significant effect on their mechanical properties. The presence of boron affected the brittleness and swelling of the matrix and the differences in mechanical properties were evident in samples exposed to temperatures above 500 °C. All six prepared composites showed tensile strength higher than 320 MPa at laboratory temperature. The boron-free composite had the highest strength 385 MPa. All samples showed a tensile strength higher than 230 MPa at elevated temperatures up to 500 °C.

## 1. Introduction 

Alkali activated aluminosilicates, called geopolymers under the certain conditions, have gained great popularity in research in recent years. The advantage of these inorganic materials is easy and fast preparation, during which it is not necessary to use a high temperature for the final hardening [1]. 

This inorganic binder based on alkali activated aluminosilicates is generally formed by mixing powdered aluminosilicates with a liquid alkaline activator. Material containing Si and Al, such as metakaolin, is usually dissolved in a liquid alkali silicate and alkali metal hydroxide solution [1,2]. The use of industrial aluminosilicate waste materials, such as coal ash and blast furnace slag, is an advantage, which leads to a reduction in the price and environmental footprint of the material [3]. The type and ratio of aluminosilicate and activator affect the resulting properties of the material [4,5,6]. Just the excellent mechanical properties, such as strength, resistance to chemicals and high temperature, is another reason for the interest in these materials [1,2,7].

Many fillers and additives can also be added to the binder, which specifically affect the properties of the resulting material [8]. Widely used fillers are sand [8,9] and fibers [10,11]. Some studies also use additives, such as compounds based on boron or phosphate [12,13,14]. Boron ions are usually represented by borax or boric acid and materials containing these in the binder are often referred to as boroaluminosilicate materials [14]. Boron is a glass-forming cation and can be four-coordinated. So, it is assumed that these characteristics can lead to improved interactions between the cation and the aluminosilicate network. The studies work with the idea that the boron ions replace the aluminum ones in the initial structure and thus the mechanism of boroaluminosilicates formation is similar to formation of pure aluminosilicates. Some authors report an increase in the strength of composites after the addition of boron to the matrix [13,14,15,16].

The use of alkali activated aluminosilicates is focused primarily on construction industry [17,18] or the immobilization of hazardous waste [19,20]. Recently, it has also been studied as a matrix of composite laminate [7,11]. Commonly used organic material matrices have a very high strength, the disadvantage is that the organic matrix (e.g., epoxy resin) cannot be used at temperatures above 300 °C [11]. Inorganic materials based on alkali activated aluminosilicates are resistant to the temperatures up to 1000 °C with no emission of toxic fumes when heated [21], including the composites with carbon fibers [22].

Under the laboratory conditions, composite laminate with inorganic matrix is often produced by applying the matrix simultaneously with the layering of fibers, roving, or fabrics. Another option is production of composite laminate with the assistance of prepregs. Thus, first the matrix is applied to the fabric, then pre-impregnated fabrics (prepregs) are stored and used to prepare composites after a certain time. Alkali activated aluminosilicates mature under normal air conditions for about 24 hours. Therefore, the composition of these materials must be adjusted to prevent hardening. Modified material used in this study can be stored at −18 °C. At this temperature, the matrix does not harden during the first month.

In this article, the influence of boron content in a matrix based on alkali-activated aluminosilicates on the mechanical properties of composites is studied. Carbon fabrics and six matrices with different boron contents were used to prepare composite plates, which were then tested for tensile strength, interlaminar strength and Young’s modulus. The tests were performed on samples cured at room conditions (ca 25 °C) and on samples heated to 400 °C, 500 °C, 600 °C and 800 °C for 1 h. Interlaminar strength (short-beam test) was also measured during the heating of samples.

## 2. Materials and Methods 

### 2.1. Materials

For preparation of alkali activated aluminosilicate matrix (A-matrix) were used commercial metakaolinite-rich material produced by the calcination of kaolinitic claystone [23,24,25] in a rotary kiln at ca 750 °C (České lupkové závody, a.s., Nové Strašecí, Czech Republic), silica fume (České lupkové závody, a.s., Nové Strašecí, Czech Republic), commercial potassium water glass with molar ratio SiO_2_:M_2_O equal to 1.7 (Vodní sklo, a.s., Prague, Czech Republic), potassium hydroxide flakes (Lach-Ner, s.r.o., Neratovice, Czech Republic), boric acid (Penta, s.r.o., Prague, Czech Republic), and distilled water. Composites were prepared from A-matrix and carbon plain weave fabric with the area weights of 200 g/m^2^ (Carbon fabric eSpread 200 CHT, Porcher Industries, La Voulte-sur-Rhône, France). The carbon fabric was chosen as the reinforcement due to the high alkalinity of the matrix. Although carbon fibers degrade at high temperatures in an oxidizing atmosphere, the use of materials such as glass or basalt in such an alkaline matrix is unsuitable.

**Figure 1:**
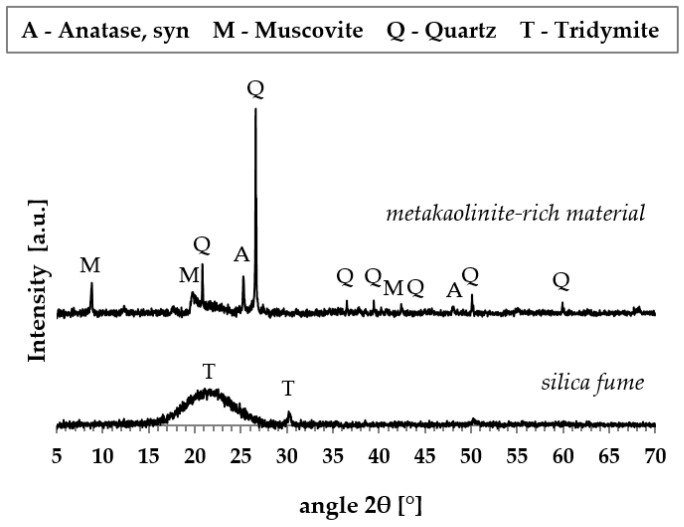
X-ray powder diffraction analysis of metakaolinite-rich and silica material.

**Figure 2:**
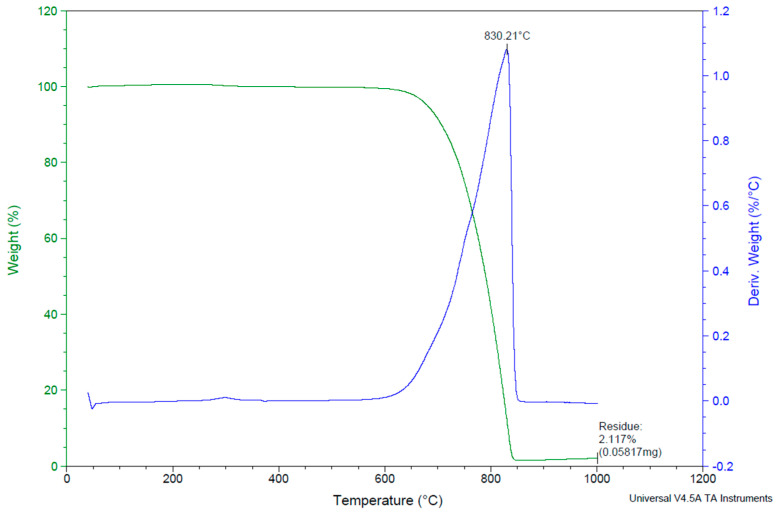
The thermal analysis curve describing the degradation of carbon fibers in the air atmosphere.

The conventional acid-base titration method and an inductively coupled plasma optical emission spectrometer OPTIMA 8000 (PerkinElmer, Waltham, MA, USA) were used for the chemical analysis of water glass while powdered materials were analyzed by X-ray fluorescence (XRF, Bruker S8 Tiger, Billerica, MA, USA). The chemical compositions of the raw materials are given in Table 1. The results of X-ray diffraction analysis (XRD, Bruker D8 Advanced, Billerica, MA, USA) are shown in Figure 1. The thermal analysis of the carbon fabric (TG/DTA – thermogravimetry/differential thermal analysis) was performed using a Discovery Series thermal analysis system (TA Instruments, New Castle, DE, USA). The sample was heated at a heating rate of 10 °C/min in flowing air (Figure 2).

### 2.2. Matrix

Starting alkaline activator A_0_ was prepared with composition of molar ratio SiO_2_/K_2_O = 1.15 by mixing the commercial potassium water glass, potassium hydroxide solution (KOH:H_2_O = 1:1 in weight ratio), and distilled water. After mixing, five different batches of solid boric acid were added to the activator A_0_ so that the resulting amounts of boric acid in the newly formed activators were 0.8%, 4%, 8%, 12% and 16%. Six activators, including reference activator A_0_, were labeled according to the amount of boric acid: A_0_, A_0.8_, A_4_, A_8_, A_12_ and A_16_. Activators were mixed in a blender (Kenwood KVL8400S Chef XL Titanium, Havant, UK) for 24 hours and stored in the fridge at 5 °C for one day. Then, the metakaolinite-rich material and silica fume were added to the alkaline activators to form six matrices named after the activators (M_0_, M_0.8_, M_4_, M_8_, M_12_ and M_16_). This mixtures of matrix with composition of SiO_2_/Al_2_O_3_ = 33.9, K_2_O/Al_2_O_3_ = 3.98, H_2_O/K_2_O = 12.1 (molar ratio) was blended ca 30 min, stored in a freezer at −18 °C for 24 h. This matrix composition was designed and verified in previous studies [12,26]. 

### 2.3. Laminate Composites

Matrices M_0 – 16_ were used to prepare six-layer composite plates with analogous names C_0_, C_0.8_, C_4_, C_8_, C_12_ and C_16_. Matrices were applied to 50 × 30 cm carbon fabric pieces using a paint roller, then the impregnated fabrics (prepregs) were individually placed between two pieces of plastic foil and stored in a freezer at −18 °C. After one week, the prepregs were taken out of the freezer, stripped of plastic foil and stacked one by one (always in the same direction) to get the composite plate. Every prepared composite plate was placed between two pieces of peel-ply fabric, wrapped in a plastic foil, compressed at 440 kPa for one hour and then cured in the oven at 65 °C for 3 h. After this time, the plates were unwrapped from the plastic foil and peel-ply fabric and finally cured for 28 days at laboratory conditions [26]. 

### 2.4. Pyrometric Cone Refractoriness

Refractoriness of the prepared matrices was obtained by a method for determining the refractory conformity of refractory raw materials with pyrometric reference cones according to the European standard EN 993-12. A heat microscope (Clasic CZ, type 0116 VAK, Řevnice, Czech Republic), pyrometric reference cones and test cones (30 × 8.5 mm, prepared according to EN 993-13) were used to the determination of matrix refractoriness. During heating (5 °C/min), the tested samples were observed and compared with the pyrometric reference cones. The results were verified by three measurements. 

### 2.5. Tensile Strength and Young’s Modulus

Prepared six-layer composite plates were cut into 250 × 25 mm samples by water jets. The ends of the samples were coated with epoxy resin and covered with sandpaper to protect the sample surface from sharp grips. Tensile strength and Young’s modulus were measured on samples stored at room temperature (ca 25 °C) and on samples that were treated with temperatures of 400 °C, 500 °C, 600 °C and 800 °C for one hour using the universal testing machine LabTest 6.200 (LaborTech, s.r.o., Opava, Czech Republic) complying with ASTM D3039. The treatment temperature was added to the name of the composites (C_0_-25, C_0_-400, C_0_-500, C_0_-600, C_0_-800, etc.).

### 2.6. Interlaminar Strength (Short-Beam Test)

Short-beam strength test was measured on samples prepared from 12 layers of carbon fabrics in the same way as the six-layer tensile strength samples. A large number of layers was used to comply with conditions of the standard ASTM D2344. Prepared samples 4 mm × 8 mm × 24 mm were tested by the universal testing machine LabTest 6.200 (LaborTech, s.r.o., Opava, Czech Republic) with a three-point bending loading configuration at a rate of crosshead speed of 1 mm/min.

### 2.7. Scanning Electron Microscope

The saturation of the carbon fabric with the matrix and changes in the morphology of the composites affected by different amounts of boric acid in the aluminosilicate matrix and by the action of different high temperatures on the composite were monitored by scanning electron microscope (SEM, JEOL JSM-IT500HR, Tokyo, Japan). Samples were coated with about 5 nm of gold, to make them conductive and to prevent charging of the samples in the microscope.

## 3. Results and Discussion

### 3.1. Pyrometric Cone Refractoriness

**Figure 3:**
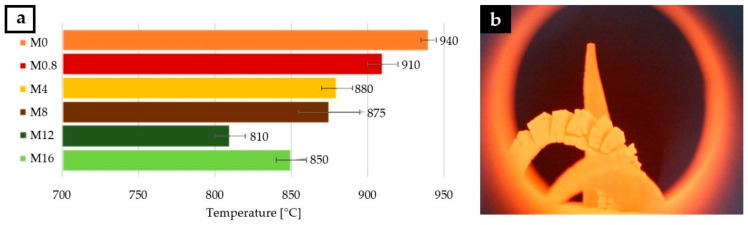
(**a**) Pyrometric refractoriness of six matrices with different content of boric acid and (**b**) tested matrix cone between two reference cones.

The results of refractoriness for the six prepared matrices are shown in Figure 3a. The pyrometric cone refractoriness of matrices was determined by simultaneously melting of two reference cones with different melting point and tested matrix cone placed in the middle (Figure 3b). The results show that all prepared matrices melt at a temperature higher than 800 °C, so the matrices are suitable for high temperature applications. Especially matrix without boric acid (M_0_) melted at a temperature of up to 940 °C. The high temperature caused a visible foaming of the tested cones. The addition of boric acid led to a reduction in the refractoriness, but also to a reduction in the undesired swelling of the matrix.

It is known that materials containing elemental Si, such as silica fume used for preparation of the matrix, may be used as a gas-forming agent at elevated temperature, thereby causing the geopolymer to swell [27,28,29]. Incorporation of boron into the aluminosilicate network [13,15] can suppress this effect [13]. However, the effect of matrix expansion can have a positive effect in applications where it is necessary to provide an increase in thermal insulation capabilities. After heating the aluminosilicate matrix without boron and with lower boron content, the porosity increases [27,28,29].

### 3.2. Expansion of Aluminosilicate Laminate Composite

An expansion of the matrix volume was also observed for laminate composite samples after heat treatment. As in the case of refractoriness measurements, this effect decreased with increasing boric acid content in the matrix. In the case of laminate composites with borosilicate matrix, the effect of sample expansion is suppressed at boric acid content higher than 4 wt. % in the alkali activator (ca 2 wt. % in matrix). Composite sample C_4_ showed the highest change in sample thickness up to 66% at temperature 500 °C. Compared to that, composite sample C_16_ had a volume change only 5% at 500 °C. 

Foaming of geopolymer with high SiO_2_ content (>50 wt. %) is described by E. Prud’homme at al. [27]. The authors describe the possibilities of the formation of foamed aluminosilicates, which they are the production of a gas (H_2_), an increase of the viscosity and a consolidation of material. Hydrogen is produced (Equation (1)) by water reduction and by the oxidation of silicon in a basic environment at temperatures above 70 °C.

4H_2_O + Si^0^ → 2H_2_ + Si(OH)_4._ (1)

**Figure 4:**
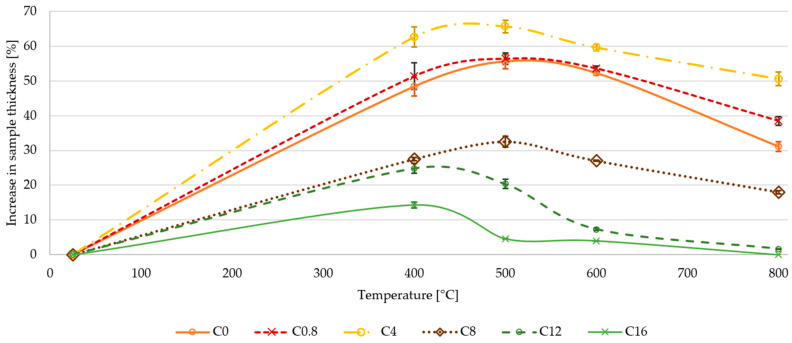
Percentage increase in sample thickness due to matrix swelling.

Thus, the addition of boric acid reduces the foaming of the matrix. These changes may be due to reduction in the percentage of silicon in the matrix, the incorporation of boron into the aluminosilicate structure, or the acidification of the basic activator with acid. Figure 4 graphically shows the percentage increase in sample volumes after heat exposure. The thickness of the samples was measured by a caliper after cooling the samples down to the room temperature. 

The tensile strength, Young’s modulus and interlaminar strength results (in MPa) were related to the original dimensions of the samples, (before the dimensional changes caused by high temperatures). This process was chosen due to the practical use of composites, especially in terms of design of construction before thermal exposure.

### 3.3. Tensile Strength and Young’s Modulus

**Figure 5:**
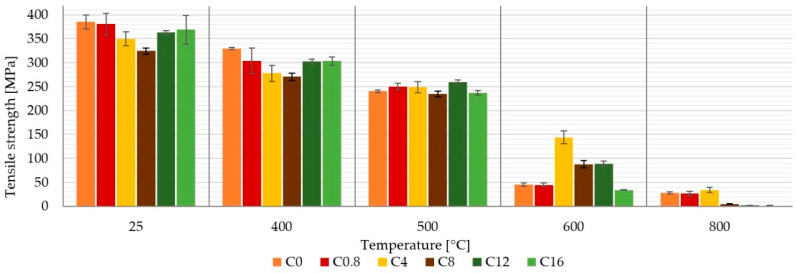
Influence of temperature and boron content in the matrix on the tensile strength of composites.

It can be seen in Figure 5 that the boron content in the matrix did not have a significant effect on the tensile strength of the laminates up to a temperature of 500 °C. This correlates with the other literature, where the effect of boron on the mechanical properties of composites is strongly dependent on the composition of the matrix binder [14,15]. The author’s results show the effect of boron depending on the content of fly, slug or sodium silicate. 

Samples without high temperature treatment had tensile strength from 385 to 324 MPa. These strengths correspond to the tensile strength of conventional duralumin. After one-hour exposition of the samples to temperatures of 400 °C and 500 °C, the samples still had very high tensile strength 329–270 MPa, 259–239 MPa, respectively. At these temperatures, laminate composites with an organic matrix are not useable. In addition, heating or fire of epoxy resin (the most common organic matrix) leads to the release of toxic and irritating gases and vapors.

At higher temperatures, there was a sharp decrease in tensile strength of composites with matrices with and without boron content. This was probably due to the thermal degradation of carbon fibers in the oxidizing atmosphere, which takes place at this temperature. The best results after exposure to 600 °C/1 h were achieved in case of composite C_4_, which also corresponds to the composition of the matrix with the largest increase in volume, as discussed earlier. After one hour at the temperature of 600 °C, this sample reached a tensile strength of 144 MPa and after exposure to temperature 800 °C/1 h, it was 34 MPa.

**Figure 6:**
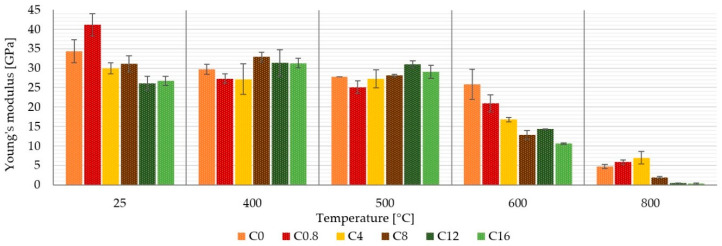
Influence of temperature and boron content in the matrix on the Young’s modulus of composites.

Figure 6 shows the effect of temperature and matrix composition on the stiffness of a laminate with an inorganic matrix. It is clear that the different boron content in the matrix did not have a significant effect on the Young’s modulus up to a temperature of 500 °C, which was mainly influenced by the stiffness of the carbon fibers. Up to these temperatures, the modulus reached values around 30 GPa. A significant decrease of the stiffness of the samples occurred at the temperature 600 °C or higher. At 600 °C, a loss of the stiffness can be seen depending on the boron content in the matrix. The higher the amount of boron, the lower the stiffness of the samples. This was probably due to the effect of boron on the reduction of the melting temperature of the binder (Figure 3a) and, thus, its higher degradation in samples with a higher boron content.

### 3.4. Interlaminar Strength (Short-Beam Test)

During the measurement of the tensile strengths of the laminates, the mechanical properties of used fabric were manifested, primarily because the loading force acted in the direction of the fibers. In the short-beam test, when the adhesion of individual layers (interlaminar strength) is determined, the force acts perpendicular to the direction of the fibers and therefore the properties of the matrix, and its adhesion to the reinforcement are more pronounced here. 

**Figure 7:**
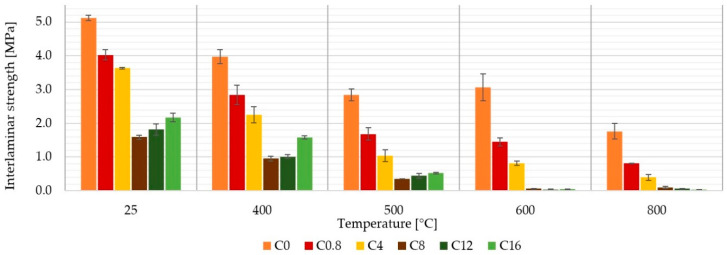
Influence of boron content in the matrix on the interlaminar strength of composites.

From the results shown in Figure 7, it is evident that the boron content has a negative effect on the interlaminar strength of the aluminosilicate matrix, which is based on clay shell, silica and carbon fibers. The interlaminar strength first decreased with the addition of boron. The lowest value of strength was shown by the C_8_ composite, the composition of which seems to be the least suitable. With the further addition of boron, the interlaminar strength increased again, but only up to a temperature of 500 °C. High temperature resistance is a significant advantage of the composites investigated in this work. The reduction in strength of samples with a higher boron content affected by a temperature higher than 500 °C is therefore very undesirable, and the effect of the addition of boron to the matrix appears to be negative.

**Figure 8:**
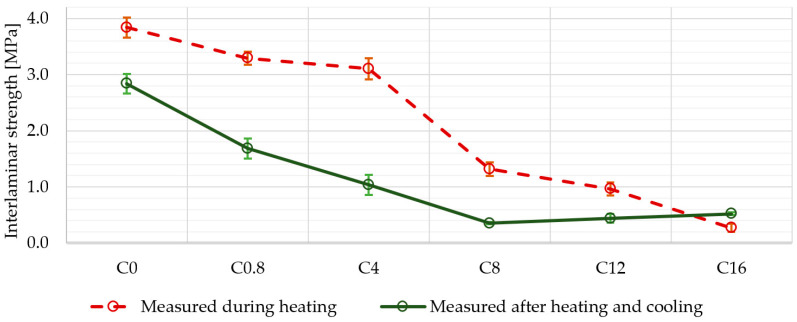
Comparison of interlaminar strength results of samples measured during and after heat treatment.

Figure 7 shows the interlaminar strength of the samples after exposing the samples to the appropriate temperature for 1 h. In contrast, interlaminar strength was also measured directly at the temperature 500 °C. Except for the sample with the highest boron content in the matrix (C_16_), a higher interlaminar strength was always found when measured at elevated temperature (Figure 8).

This was probably due to the fact that the sample was cooled after high temperature and the matrix became brittle due to volume changes. As the temperature rises, the plasticity of the matrix material also appears to increase and thus its stiffness decreases. However, it was not possible to perform tensile tests at higher temperatures, so unfortunately, we do not have this data available, and we can only deduce it from the results obtained in the graph in Figure 8. The increased plasticity and, thus, the reduced brittleness of the matrix aids the mutual adhesion of the individual layers and therefore the measurements performed at elevated temperature showed a higher interlaminar strength than after being cooled down.

### 3.5. Scanning Electron Microscope

**Figure 9:**
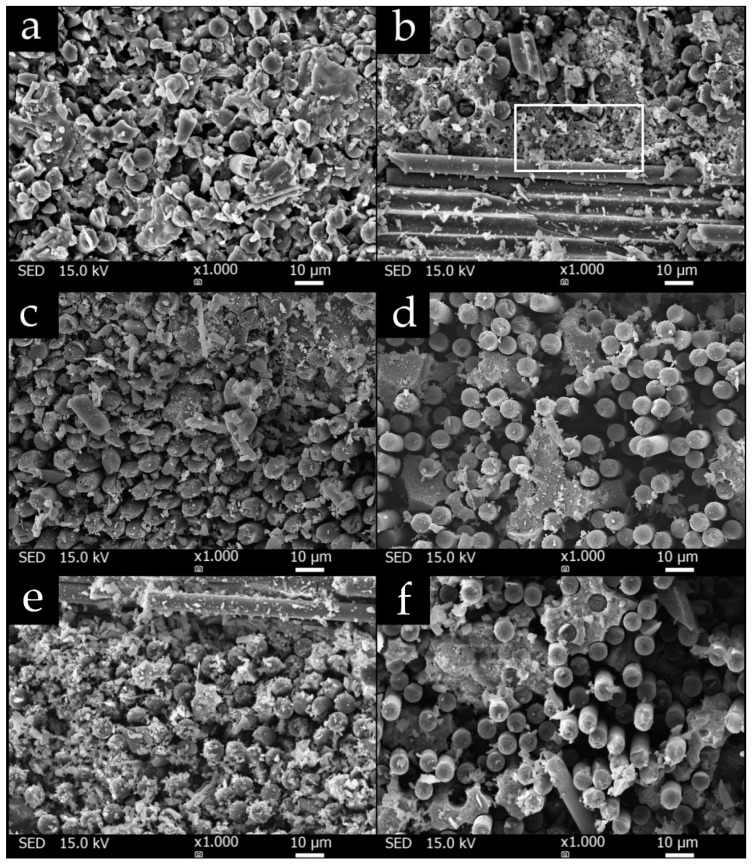
Photos of composite samples (**a**) C_0_-25, (**b**) C_0_-500, (**c**) C_8_-25, (**d**) C_8_-500, (**e**) C_16_-25, (**f**) C_16_-500 obtained using scanning electron microscope.

SEM photos showed that the fabrics were very well saturated with matrix in all prepared composite plates. Figure 9 shows SEM photos of a composite with a matrix without boric acid C_0_ (Figure 9a,b), composite C_8_ (Figure 9c,d) and composite with a matrix with the highest amount of boric acid C_16_ (Figure 9e,f). There is a clearly visible difference between the matrices. Matrix of C_0_-25 composite (Figure 9a) is smooth and fused which confirms the toughness of the sample, while, in the case of the C_16_-25 composite (Figure 9e), a fragmented matrix can be seen, which has become brittle due to the presence of boron. As a result of the embrittlement of the matrix, the matrix was more often chipped out of the composite. This phenomenon was also observed while cutting the composite to obtain a smaller sample that could be placed in the SEM. Furthermore, we can see in Figure 9 that the matrix of the C_0_ composite became porous after a temperature of 500 °C (Figure 9b—the pores are visible in the white border area), while the increased boron content of the C_16_ sample significantly reduced the porosity (Figure 9f).

Differences in the interface between the fibers and the matrix are also visible in the presented SEM photos. Thus, the addition of boron to the matrix appears to affect the adhesion of the fiber to the matrix, which may have caused differences in interlaminar strength. This supports SEM photo of the C_8_ composite (Figure 9c,d), where a significant decrease in the adhesion of the matrix to the fibers is seen. It was sample C_8_ that showed the lowest interlaminar strength. The results show that the composition of the matrix M_8_ is the least suitable for the preparation of composites, and that with an increasing amount of boron, the strength can increase again.

## 4. Conclusions

This paper presents a comparison of carbon composites prepared from six inorganic matrices differing in boric acid content. The composites were made of carbon plain wave fabrics and aluminosilicate matrices in prepreg form. The obtained results can be drawn in the following conclusions:

- A boric acid content of up to 16 wt. % in the alkaline activator (approximately 8 wt. % in the matrix) has not been shown to be significantly suitable for improving the mechanical properties of aluminosilicate laminates prepared by the prepreg method.
- The boric acid content affected the behavior of the matrix. The higher boron content reduced the swelling of the matrix when heated to higher temperatures, but caused its embrittlement.
- Up to a temperature of 500 °C, all samples were relatively stable and always showed a tensile strength higher than 230 MPa.
- Without temperature treatment, the highest tensile strength showed samples without boron content C_0_ (385 ± 14 MPa) and the lowest tensile strength showed samples C_8_ (324 ± 6 MPa).
- Laminate C_4_ with the biggest volume change had the highest tensile strength (144 ± 13 MPa) after 600 °C.
- Up to 500 °C, Young’s modulus was most affected by the properties of the reinforcement (carbon fiber) and by the boron content at temperature above 600 °C.
- Boron content had a negative effect on interlaminar strength.

## Figures and Tables

**Table 1:** Chemical composition of raw materials.

Material	Material Composition (%)								
	H_2_O	SiO_2_	Al_2_O_3_	Na_2_O	K_2_O	CaO	P_2_O_5_	Fe_2_O_3_	ZrO_2_
Potassium water glass	63.9	18.9		0.23	16.7				
Metakaolinite-rich material	1.69	52.8	41.7		0.84	0.16	0.08	0.92	
Silica fume	0.99	96.4	0.42		0.04	0.09	0.42		1.27
